# Nano-synthesis of solid acid catalysts from waste-iron-filling for biodiesel production using high free fatty acid waste cooking oil

**DOI:** 10.1038/s41598-020-70025-x

**Published:** 2020-08-06

**Authors:** E. O. Ajala, M. A. Ajala, I. K. Ayinla, A. D. Sonusi, S. E. Fanodun

**Affiliations:** 1 Department of Chemical Engineering, University of Ilorin, Ilorin, Nigeria; 2 Department of Industrial Chemistry, University of Ilorin, Ilorin, Nigeria

**Keywords:** Chemistry, Energy science and technology, Engineering, Materials science, Nanoscience and technology

## Abstract

Waste-iron-filling (WIF) served as a precursor to synthesize α-${\text{Fe}}_{2} {\text{O}}_{3}$ through the co-precipitation process. The α-${\text{Fe}}_{2} {\text{O}}_{3}$ was converted to solid acid catalysts of RBC500, RBC700, and RBC900 by calcination with temperatures of 500, 700 and 900 °C respectively and afterwards sulfonated. Among the various techniques employed to characterize the catalysts is Fourier transforms infrared spectrometer (FT-IR), X-ray diffraction (XRD and Scanning electron microscopy (SEM). Performance of the catalysts was also investigated for biodiesel production using waste cooking oil (WCO) of 6.1% free fatty acid. The XRD reveals that each of the catalysts composed of Al–${\text{Fe}}_{2} {\text{O}}_{3} /{\text{SO}}_{4}$. While the FT-IR confirmed acid loading by the presence of ${\text{SO}}_{4}^{2 - }$ groups. The RBC500, RBC700, and RBC900 possessed suitable morphology with an average particle size of 259.6, 169.5 and 95.62 nm respectively. The RBC500, RBC700, and RBC900 achieved biodiesel yield of 87, 90 and 92% respectively, at the process conditions of 3 h reaction time, 12:1 MeOH: WCO molar ratio, 6 wt% catalyst loading and 80 °C temperature. The catalysts showed the effectiveness and relative stability for WCO trans-esterification over 3 cycles. The novelty, therefore, is the synthesis of nano-solid acid catalyst from WIF, which is cheaper and could serve as an alternative source for the ferric compound.

## Introduction

Recently, the spotlight is on bioenergy with attendants scientific research due to peculiar challenges in the energy industry such as increased energy demand and energy insecurity with increasing concern for fossil fuel^1^. Bioenergy such as biodiesel which is also known as fatty acid methyl esters (FAME) is receiving worldwide attention as a substitute for fossil fuel because it is non-toxic, biodegradable, has negligible sulfur and greenhouse emissions^2^. However, biodiesel production worldwide is challenged by its competition with food crops, which is the main feedstock for its production and the cost of catalyst^1^. The production of biodiesel has 70.6% of its cost on food crop as feedstock, 12.6% on chemicals for catalyst synthesis while energy, maintenance, labor, and depreciation takes 16.8%^3^. Therefore, suitable and cheap feedstock such as waste cooking oil (WCO) other than food crops is essential as raw material for sustainable biodiesel production^4^.

The WCO is reported to be highly available all over the world and can generate about 0.095 million tons of biodiesel. It can serve as a feedstock in biodiesel production, instead of its illegal dumping into landfills and rivers, thereby causing significant environmental pollution^3^. Thus, it will not compete with food crops in this context but solve an environmental challenge^4^. Nevertheless, its challenges in biodiesel production are the presence of high free fatty acid (FFA) and moisture content which result in soap formation, difficult operation and separation of product from the catalyst, together with low yield and high cost of production when a homogeneous catalyst is used^4,5^. Therefore, the need for heterogeneous catalysts is now of great concern among researchers.

Heterogeneous (solid) catalyst, either acid or base type, is environment-friendly with a good quality yield of biodiesel. It is cheap to synthesize, can easily be recovered and reused. Also, solid catalyst eliminates separation, emulsification and soap production challenges associated with homogeneous catalysts^1^. Meanwhile, the solid acid catalyst is more suitable for high FFA feedstock in simultaneous esterification and transesterification reaction using a one-pot process to produce FAME^6^. Other advantages of the solid acid catalyst include the elimination of washing stage, easy reactivation, selectivity, corrosion-free and improved product purity^7^. Despite these numerous advantages associated with the use of the solid acid catalyst for biodiesel production, there are some drawbacks. The catalytic performances of the catalyst in the transesterification process are not satisfactory, as there is serious leaching of active species into the reaction mixture which leads to great deactivation of the catalysts. However, efforts are ongoing to develop a robust and efficient solid catalyst for trans-esterification of low-cost vegetable oils/animal fats for biodiesel production in a one-pot synthesis which would help to improve the economic feasibility of biodiesel to replace fossil-fuel^8^. Researchers have used oxides of different metals as support for the development of solid acid catalyst^9^. These catalysts are: ${\text{Nb}}_{2} {\text{O}}_{5} /{\text{SO}}_{4}$, ${\text{SO}}_{4}^{2 - } /{\text{ZrO}}_{2}$, ${\text{SO}}_{4}^{2 - } /{\text{SnO}}_{2}$–SiO_2_, ${\text{SO}}_{4}^{2 - } /{\text{TiO}}_{2}$–SiO_2_ and ${\text{SO}}_{4}^{2 - } /{\text{ZrO}}_{2}$–Al_2_O_3_^10,11^_._ Others include ${\text{FeH}}_{28} {\text{NO}}_{20} {\text{S}}_{2}$, Fe_2_O_3_–MnO–${\text{SO}}_{4}^{2 - } /{\text{ZrO}}_{2}^{2}$, ${\text{Fe}}_{2} \left( {{\text{SO}}_{4} } \right)_{3} /{\text{C}}$, ${\text{Fe}}_{2} {\text{SO}}_{4}$, ${\text{CaO}}/{\text{Fe}}_{2} \left( {{\text{SO}}_{4} } \right)_{3}$ and Li–${\text{CaO}}/{\text{Fe}}_{2} \left( {{\text{SO}}_{4} } \right)_{3}$^12,13^. Although, ferric oxide compound has been reported for solid acid catalyst production but can also be developed into non-acid solid catalyst such as ${\text{Fe}}/{\text{C}}$, α-${\text{Fe}}_{2} {\text{O}}_{3}$, CaFe_2_O_4_–${\text{Ca}}_{2} {\text{Fe}}_{2} {\text{O}}_{5}$, ${\text{MgFe}}_{2} {\text{O}}_{4} @{\text{CaO}}$ and CaO–γ–${\text{Fe}}_{2} {\text{O}}_{3}$^1,5,9,14,15^. These show that the ferric compounds are highly suitable to develop various catalysts for biodiesel production. Widayat Widayat et al.^16^ synthesized hematite $\left( { \propto {\text{-} \text{ Fe}}_{2} {\text{O}}_{3} } \right)$ magnetic nanoparticles to produce biodiesel through the trans-esterification process. The percentage yield of biodiesel obtained was 86.78% with FAME content of 87.88%. In their study, the hematite was obtained from iron sand and was not sulphated. A magnetic solid acid catalyst, consisting of a core of iron oxide nanoparticles, a poly(glycidyl methacrylate) shell, and sulfonic acid groups on the surface, was synthesized by Zillillah et al.^17^. The catalyst was used in an esterification reaction of grease (FFA = 16 wt%) with methanol to produce biodiesel yield of 96%. Though the catalyst was reported to be highly active, stable and recyclable, it is likely to be very expensive as it is a combination of two synthetic chemical substances. Guanidine-functionalized ${\text{Fe}}_{3} {\text{O}}_{4}$ magnetic catalyst for biodiesel production was reported in literature^18^. The catalyst was synthesized through three steps, (1) co-precipitation of Fe(II) and Fe(III) ions, (2) surface modification with chloropropyl groups and (3) functionalization with guanidine. The protocol of synthesis is a too cumbersome process and analytical grade of a synthetic chemical substance was used to functionalize the catalyst. Also, a magnetic solid acid catalyst of ${\text{S}}_{2} {\text{O}}_{8}^{2 - } /{\text{ZrO}}_{2}$–TiO_2_–Fe_3_O_4_ was developed for biodiesel production^19^. The study also shows that the analytical grade of ${\text{FeSO}}_{4} \cdot 7{\text{H}}_{2} {\text{O}}$ and ${\text{Fe}}_{2} \left( {{\text{SO}}_{4} } \right)_{3}$ were used as precursors to synthesized ${\text{Fe}}_{3} {\text{O}}_{4}$ which was further impregnated with ${\text{ZrO}}_{2}$ and ${\text{TiO}}_{2}$ to functionalized the catalyst. Xie et al.^20^ utilized ${\text{Fe}}_{3} {\text{O}}_{4}$ composite-supported sodium silicate as heterogeneous catalyst for biodiesel production. A crystalline ${\text{Fe}}/{\text{Fe}}_{3} {\text{O}}_{4}$ core/shell magnetic catalyst that was synthesized for biodiesel production was reported to have excellent stability^21^. The catalysts were coated with silica after functionalizing them with either sulfamic acid or sulfonic acid. These findings in literature justify that the catalysis of magnetic nanoparticles are interesting and versatile materials. This is due to its high surface area-to-volume ratio, which facilitates surface modification. Studies have shown that the magnetic-based solid acid catalyst has more catalytic activity compare to traditional acid catalysts which is due to the magnetic attraction that provides strong ionic interaction between the particles, leading to high catalyst activity and stability^22^. Hence, magnetic materials are alternative support material for catalyst development in biodiesel production, as it is easy to synthesize and functionalize, cheap to produce, low toxicity and easy to recover^18^. Even though, leaching of the active specie in magnetic nanocatalysts of trans-esterification reaction is a major challenge. The heterogeneity makes the separation of the catalyst easy, the leaching of active species of heteropoly acid cannot be prevented, which further leads to low reusability^23^. In magnetic nanocatalysts, the leaching which occurs on the surface is associated with a partial dissolution of the iron oxide nanoparticles^18^. It is worthy to note that all the aforementioned studies used iron sand or analytical grade of high purity (98%) ferric compound to synthesize their catalysts, in contrast to this study. This study investigates the synthesis of the magnetic-based sulfonated catalyst using waste-iron-fillings which is novel and it is expected to improve cost-saving and environmental friendliness of the catalyst for biodiesel production.

Waste-iron-filings (WIF) are very small fragments, minute pieces of iron or galvanized iron from excess steels in factories and workshops, which are harmful to the environment^24^. These wastes are increasing daily and constituting increase in environmental pollution. In 2017, global WIF was estimated at 750 MT, of which 630 MT was recycled with the remaining 120 MT (16% of total) disposed in the landfill. The future projection of WIF is about 1 billion tonnes (BT) by 2030 and expected to reach 1.3 BT by 2050. This revealed that by then, the WIF for landfill would be 0.2 BT (0.2 trillion kg)^25^. This large quantity of WIF might cause serious environmental concern if alternative use is not found. Hence, the need to find more economical use for the WIF.

This study, therefore, investigates the use of waste-iron-filling (WIF) as a cheap, novel and alternative source of a ferric compound which is to replace high expensive ferric compound (98% purity) for catalyst development in biodiesel production. The WIF was developed into hematite $\left( {\alpha {\text{-} \text{ Fe}}_{2} {\text{O}}_{3} } \right)$ and characterized for its suitability for catalyst synthesis through XRF and TG–DTA. The $\left( {\alpha {\text{-} \text{ Fe}}_{2} {\text{O}}_{3} } \right)$ was calcined at various temperatures of 500, 700 and 900 °C, and afterwards, sulfonated by ${\text{H}}_{2} {\text{SO}}_{4}$ to develop the catalysts as RBC500, RBC700 and RBC900 respectively. The catalysts were characterized by their elemental composition using XRD and EDX, micrograph (SEM), functional group (FTIR), surface area and pore volume (BET). The functionality of the catalysts for biodiesel production using high FFA WCO was also investigated. The biodiesel produced was characterized by their physicochemical properties and fatty acid methyl esters (FAME) profile.

## Materials and methods

### Chemicals and materials

Analytical grade of Sigma Aldrich reagents was used in this study. These include; methanol (MeOH), hydrogen peroxide (30% w/w, ${\text{H}}_{2} {\text{O}}_{2}$), hydrochloric acid (37% v/v, ${\text{HCl}}$), and tetraoxosulphate IV acid (98% v/v, ${\text{H}}_{2} {\text{SO}}_{4}$). The WIF was collected from the Central Engineering Workshop, University of Ilorin, Nigeria and the WCO was obtained from Item 7 restaurant, University of Ilorin, Nigeria.

### Preparation of WIF to synthesis hematite α-${\text{Fe}}_{2} {\text{O}}_{4}$

The hematite was synthesized by weighing 20 g cleaned WIF into 1 l conical flask with 200 ml of 6 M dilute ${\text{HCl}}$. The mixture was heated at 70 °C for 50 min to completely dissolve the WIF to form ${\text{FeCl}}_{2}$. At the end of the conversion to ${\text{FeCl}}_{2}$, color changes from grey to dark green was noticed with wood shavings-like particles and polymeric particles found. The reaction product was filtered with a muslin cloth and the lost volume was replaced with ${\text{HCl}}$. This process is known as co-precipitation as shown in Eq. (1).

1 $${\text{Fe}}_{\left( s \right)} + 2{\text{HCl}} \to {\text{FeCl}}_{{2 \left( {aq} \right)}} + {\text{H}}_{2 \left( g \right)}\uparrow$$

Oxidation of the ${\text{FeCl}}_{2}$ in Eq. (1) to ${\text{Fe}}\left( {{\text{OH}}} \right)_{3}$ (reddish-brown) was achieved by adding 100 ml of ${\text{H}}_{2} {\text{O}}_{2}$^26^ as shown in Eq. (2).

2 $$6{\text{FeCl}}_{{2 \left( {aq} \right)}} + 3{\text{H}}_{2} {\text{O}}_{2} \to 2{\text{Fe}}\left( {{\text{OH}}} \right)_{3} + 4{\text{FeCl}}_{3}$$

The ${\text{Fe}}\left( {{\text{OH}}} \right)_{3}$ obtained in Eq. (2) was thermally decomposed under a controlled environment of nitrogen in a furnace at 300 °C for 2 h to obtain hematite α-$({\text{Fe}}_{2} {\text{O}}_{3} )$^27^ as shown in Eq. (3).

3 $$2{\text{Fe}}\left( {{\text{OH}}} \right)_{3} \xrightarrow[{\Delta \left( {300{\kern 1pt} ^{^\circ } {\text{C}}} \right)}]{}{\text{Fe}}_{2} {\text{O}}_{3} + 3{\text{H}}_{2} {\text{O}}$$

**Figure 1:**
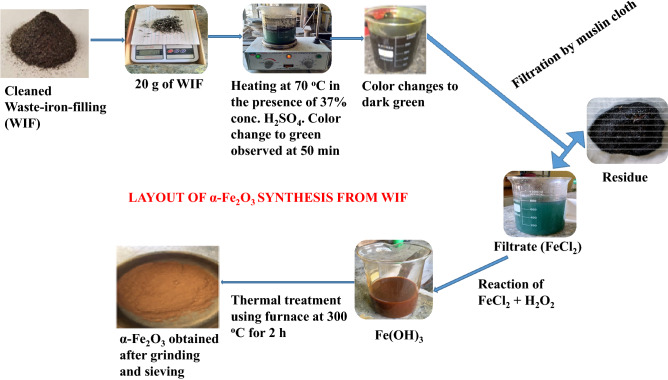
Schematic layout of hematite synthesis from WIF.

Finally, the sample was ground to a fine powder and characterized to determine its elemental compositions and thermal stability. A schematic layout showing the step-by-step approach to the synthesis of hematite from WIF is represented in Fig. 1.

### Catalyst preparation

The hematite $\left( {\alpha {\text{-} \text{ Fe}}_{2} {\text{O}}_{3} } \right)$ contaminated with alumina obtained from the WIF was calcined at 500, 700 and 900 °C according to Won Jung et al.^28^ to synthesize RBC500, RBC700 and RBC900 respectively. Thereafter, sulfonated to obtain the kind of catalysts as shown in Eq. (4).

4 $${\text{Fe}}_{2} {\text{O}}_{3} \left( {{\text{Al}}} \right) + {\text{H}}_{2} {\text{SO}}_{4} \to ({\text{Al}} - {\text{Fe}}_{2} {\text{O}}_{3} - {\text{SO}}_{4} ) + {\text{H}}_{2\left(g\right)}\uparrow$$

Thirty grams of calcined α-Fe_2_O_3_ was dissolved in 55 g of H_2_SO_4_ (in a stoichiometric ratio 1:3) using a 1 l conical flask, placed on a magnetic stirrer to continuously stir for 1 h. The obtained slurry was oven-dried at 110 °C for 2 h to remove moisture. Thereafter, the dried sample was washed by a vacuum pump filter using hot distilled water (70 °C) to remove excess H_2_SO_4_. The recovered water was continuously checked for the presence of sulfate ions using barium chloride $\left( {{\text{BaCl}}_{2} } \right)$ solution until the white precipitate disappears. The resulting sample was dried in a vacuum at 120 °C for 4 h to get the sulfated-hematite sample in the form of Al–${\text{Fe}}_{2} {\text{O}}_{3} /{\text{SO}}_{4}$ catalyst. The catalyst was ground and sieved at < 0.25 nm to obtain fine particles and thereby, identified as RBCs (RBC500, RBC700, and RBC900).

### Samples characterization and equipment

#### X-ray fluorescence (XRF)

The elemental composition of the WIF and obtained hematite were carried out using Thermo Scientific Niton (model; XL3t XRF), to determine the elements present in the WIF and ascertain the purity level of the α-Fe_2_O_3_ produced from WIF.

#### Thermogravimetry–differential thermal analysis (TG–DTA)

PerkinElmer TGA 4000 (Netherland) was used to determine the thermal stability of the α-${\text{Fe}}_{2} {\text{O}}_{3}$. While nitrogen gas was used at operating conditions of 60 ml/min flow rate and 10 °C/min heating rate from 25 to 1,000 °C for the analysis.

#### Fourier transform infrared spectrophotometer (FTIR)

The FTIR spectra of the samples were analysed by an FT-IR Shimadzu (8400S) spectrometer, to identify the functional groups present in the α-Fe_2_O3 and RBCs. The samples were pelletized with potassium bromide, and the spectra were obtained from the accumulation of 32 total scans in the range of wavenumbers 450–4,000 cm^−1^ with a resolution of 4 cm^−1^.

#### X-ray diffraction (XRD)

Crystallite size and chemical composition of the α-${\text{Fe}}_{2} {\text{O}}_{3}$ and RBCs were determined using XRD (Bruker AxSD8) by Copper (Cu)-Kα radiation diffractometer with 2θ range 20°–90°, a step size of 0.028°, operating at 45 kV and 40 mA. The crystalline sizes (D) of the samples were calculated by the Debye–Scherrer’s equation as shown in Eq. (5).

5 $$D = \frac{k\lambda }{{\beta cos\theta }}$$

where D is the crystalline size, k (constant) = 0.94, λ is the (Cu)-Kal wavelength = 1.54060, θ is Bragg angle and β is the full width at half maximum (FWHM) of the peak at 2θ.

#### High-resolution scanning electron microscopy (HRSEM) coupled with energy dispersion spectrum (EDS)

Micrographs and elemental composition of the α-${\text{Fe}}_{2} {\text{O}}_{3}$ and RBCs were observed using HRSEM coupled with EDS of Zeiss Auriga. The samples were coated with gold–palladium (Au: Pd; 60:40) using Quorum T15OT for 5 min before analysis, to prevent charging that can distort images when analyzing. The microscope was operated at 5 keV for imaging and 20 keV for the EDS detector.

#### Dynamic light scattering (DLS)

This was employed to evaluate the size distribution and average particle size of the α-Fe_2_O_3_ and RBCs samples. The DLS was performed using a Malvern Instruments-ZEN1600 (Serial number: MAL 1084260, UK) in backscattering mode at an angle of 173 ℃ and was operated at 25 °C. The results obtained were presented as size distribution by the intensity with the average particle size.

#### Nitrogen adsorption–desorption analysis

The surface area, mean pore volume and mean pore size diameter of the α-${\text{Fe}}_{2} {\text{O}}_{3}$ and RBCs were determined by a Nova 4200e Quantachrome (USA). Before sample analysis, the equipment was degassed at 250 °C under vacuum for 12 h. The sample of 120 mg was measured into a glass cell and heated at 250 °C for 3 h under vacuum to remove impurities adsorbed on the surface of the catalyst. Nitrogen adsorption isotherms were obtained to calculate the surface area, pore volume and pore diameter using the Brunauer–Emmett–Teller (BET) equation.

### Evaluations of acidic and basic strength of the RBCs

The titration procedures reported by Zhihuan Weng et al.^29^ was used to evaluate acidic and basic sites of the α-${\text{Fe}}_{2} {\text{O}}_{3}$ and RBCs. The acidic sites were determined by titrating the sample with 4 mL of 0.01 M ${\text{Na}}_{2} {\text{CO}}_{3}$ solution and filtered. Then, the supernatant was treated with 5 mL of 0.0244 M HCl solution and titrated with 0.01 M ${\text{NaOH}}$ using phenolphthalein as the indicator. The basic sites were also determined by neutralizing 25 mg of the sample with 5 mL of 0.0224 M ${\text{HCl}}$ solution. The mixture was filtered and the supernatant reacted with 0.01 M ${\text{NaOH}}$ solution in an acid–base titration using phenolphthalein as the indicator. These procedures were used to estimate the surface coverages of the acidic and basic sites of the samples^29^. The acid and basic sites concentrations obtained from the samples are the average values obtained over triplicate experimental runs.

### Simultaneous methanolysis of WCO by the RBCs

**Figure 2:**
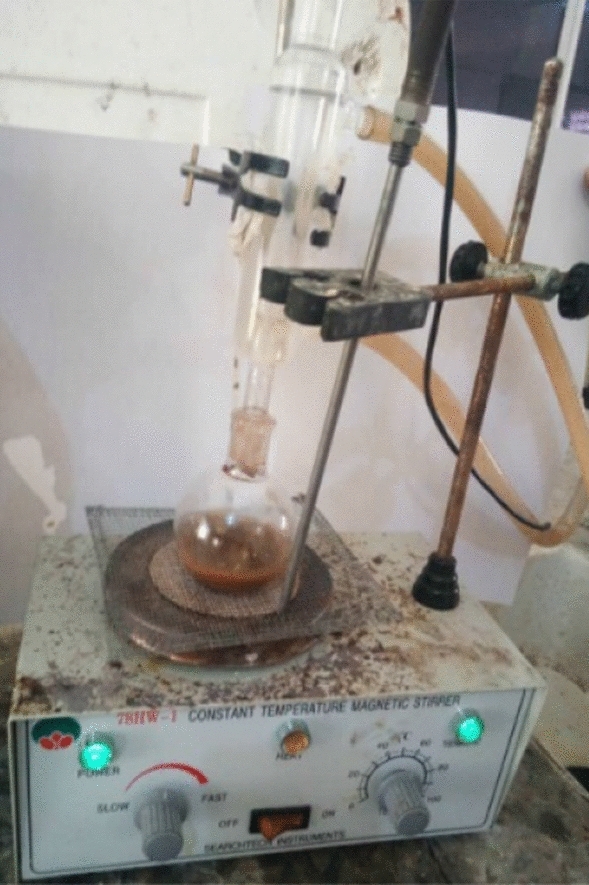
Experimental set-up.

**Figure 3:**
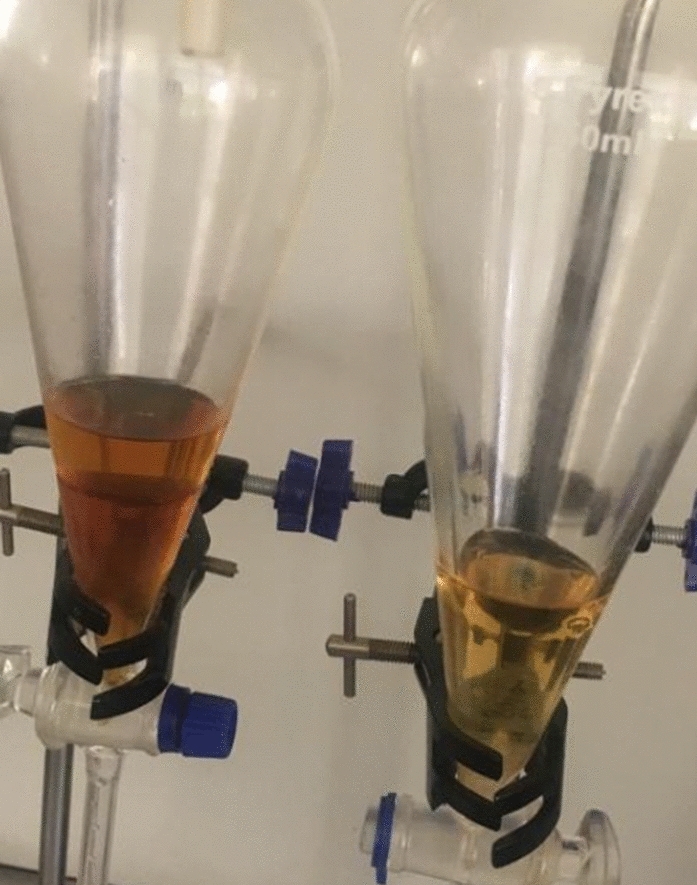
The FAME in separating funnel.

The methanolysis of WCO that contains 6.1% of FFA (12.2 mg $${\raise0.7ex\hbox{${{\text{KOH}}}$} \!\mathord{\left/ {\vphantom {{{\text{KOH}}} {\text{g}}}}\right.\kern-\nulldelimiterspace} \!\lower0.7ex\hbox{${\text{g}}$}} \; {\text{oil}}$$ of acid value) was performed in a flat bottom flask connected to a condenser for conventional normal reflux of methanol vapor as shown in Fig. 2. The process was carried out in a simultaneous reaction process of esterification and transesterification which is also known as a trans-esterification reaction, using the prepared catalysts (RBCs). The WCO of 100 g and methanol (MeOH) molar ratio 6:1 of WCO was weighed into the flask with 10 g of the catalyst and the reaction began by stirring the mixture at 800 rpm continuously and heating to 70 °C for a reaction time of 4 h using a hot-plate magnetic stirrer. After the reaction, the mixture was allowed to cool at room temperature and separated by a centrifuge at 1,500 rpm for 10 min. Excess MeOH recovery and glycerol separation were carried out in a separatory funnel as shown in Fig. 3. The crude biodiesel obtained was further purified using the method of Cholada Komintarachat and Sathaporn Chuepeng^30^. The experimental studies were investigated in triplicate and the average values were obtained with standard deviations. The percentage yield of biodiesel was determined using Eq. (6).

6 $$Biodiesel\,Yield \left( \% \right) = \frac{Weight\,of\,biodiesel}{{Weight\,of\,WCO}} \times 100$$

### Reusability study of the RBCs

One of the benefits of solid acid catalyst for biodiesel production is the ability to be re-used over some cycles. The reusability of the RBCs was also investigated by recycling the catalyst after completion of each transesterification reaction over five cycles. The solid acid catalyst was conveniently recovered by a centrifuge at 1,500 rpm for 10 min, washed thoroughly with n-hexane and methanol^31^ while the experiments were conducted at 12:1 of MeOH: WCO molar ratio, 8 wt% catalyst loading for 3 h and reaction temperature of 80 °C.

### Physico-chemical and FAME characterizations of the WCO and biodiesel by the RBCs

#### Physico-chemical properties

The physical and chemical properties of the WCO and biodiesel samples by the RBCs were evaluated using appropriate America Standard Testing Methods (ASTM). The density was studied by ASTM D5002, kinematic viscosity by ASTM D445 and flash point by ASTM D93. Other properties studied by the ASTM standard are cloud point (ASTM D2500), pour point (ASTM D97) and cetane number (ASTM D613).

#### FAME analysis

The fatty acid methyl ester (FAME) yield and profile of the WCO and biodiesel samples by the RBCs were investigated. This was however achieved by using a Gas Chromatography–Mass Spectrophotometer (GC–MS), Agilent 7890A equipped with flame ionization detector. Biodiesel sample of 250 mg was weighed into the sample bottle and placed in the sample tray. Helium was used as carrier gas with 1 mL/min constant flow with vacuum compensation through a split ratio of 30:1, and injection temperature of 250 °C. The GC–MS was operated at the temperature range of 50 to 290 °C at 3°/min and the total runtime of 62 min with the acquisition from 30 min. The FAME spectra were obtained and compared with those of the standard spectra from the NIST library (NIST 11)^32^.

## Results and discussion

### XRF of the WIF, hematite obtained from WIF and ore

Table 1 shows the elemental composition of the WIF and α-${\text{Fe}}_{2} {\text{O}}_{3}$ obtained in this study and α-${\text{Fe}}_{2} {\text{O}}_{3}$ reported by Abraham Muwanguzi et al.^33^. The composition of the WIF shows that the ${\text{Fe}}_{2} {\text{O}}_{3}$ (68.6) has the highest concentration, follow by ${\text{Al}}_{2} {\text{O}}_{3}$ (14.21). This result reveals that the WIF contains in high concentration, two amphoteric oxides suitable as a catalyst for biodiesel production. The processed WIF reveals the presence of α-${\text{Fe}}_{2} {\text{O}}_{3}$ (74.43%), ${\text{Al}}_{2} {\text{O}}_{3}$ (12%), ${\text{MgO}}$ (7.02) and ${\text{CaO}}$ (4.10) as the major elements, while other associated elements which include ${\text{CuO }}$ (0.36%), ${\text{MnO}}$ (0.39%), ${\text{Cr}}_{2} {\text{O}}_{3}$ (0.30%), ${\text{TiO}}_{2}$ (0.01), ${\text{ZnO}}$ (0.63%), ${\text{SiO}}_{2}$ (0.87), ${\text{Rb}}_{2} {\text{O}}$ (0.02) and ${\text{Eu}}_{2} {\text{O}}_{3}$ (0.68) exist as traces in < 0.1%. This results revealed that the product of the WIF essentially contains α-${\text{Fe}}_{2} {\text{O}}_{3}$ and ${\text{Al}}_{2} {\text{O}}_{3}$ with fewer impurities which is similar to the results obtained from the hematite ore (α-${\text{Fe}}_{2} {\text{O}}_{3}$ (92.6%), and ${\text{Al}}_{2} {\text{O}}_{3}$ (1.35%) reported by Abraham Muwanguzi et al.^33^. Therefore, hematite synthesized from WIF by co-precipitation is an alternative to commercial ${\text{Fe}}_{2} {\text{O}}_{3}$.

**Table 1:** XRF analysis of the WIF and hematite $\alpha { - }\left( {{\text{Fe}}_{2} {\text{O}}_{3} } \right)$.

S/N	Compound	WIF	α-${\text{Fe}}_{2} {\text{O}}_{3}$ (%)	
1	Fe_2_O_3_	68.6	74.43	92.6
2	Al_2_O_3_	14.21	12.00	1.35
3	MnO	0.99	0.39	0.01
4	CuO	0.91	0.36	< 0.01
5	Cr_2_O_3_	0.45	0.30	–
6	TiO_2_	0.09	0.01	0.07
7	ZnO	1.20	0.63	< 0.01
8	SiO_2_	1.79	0.87	2.28
9	CaO	0.60	4.10	–
10	Rb_2_O	0.07	0.02	–
11	MgO	10.02	7.02	–
12	Eu_2_O_3_	1.0	0.68	–
13	SrO	0.22	LOD	–
14	Y_2_O_3_	0.45	LOD	–
Source		This study	This study	Abraham Muwanguzi^35^

### TG–DTA of the hematite

**Figure 4:**
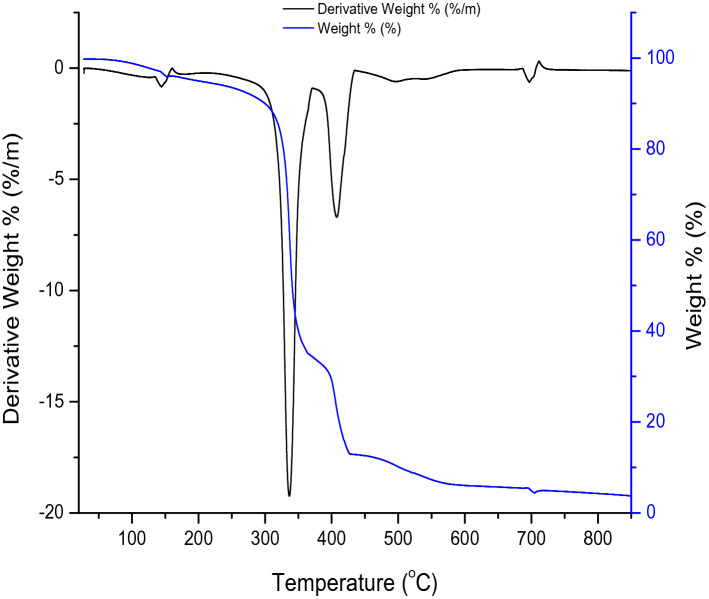
TGA/DTA curves of the hematite.

Thermal stability of the α-${\text{Fe}}_{2} {\text{O}}_{3}$ by the TG–DTA analyses are shown in Fig. 4**.** The thermograph shows three main regions of decomposition. At a lower temperature of about 300 °C, weight loss of 10 wt% was observed which was due to the breaking of weakly bonded water molecules by physio-sorption^6^. At an elevated temperature of between 300 and 500 °C, weight loss of 55 wt% and 25 wt% from 320 to 370 °C and 370 to 420 °C occurred respectively. These might be due to the condensation of sinol group (Si–O) compound present in the hematite. Although 10 wt% weight loss was recorded between 420 and 600 °C due to the decomposition of chemical components thus showing crystalline phase transformation^1^. The stability of the weight was experienced at a temperature above 500 °C which indicates that the α-${\text{Fe}}_{2} {\text{O}}_{3}$ synthesized remains stable at that temperature and corroborates with the purity nature of the sample. Hence, the three main regions observed are physically adsorbed water, removal of chemically adsorbed water, and decomposition of chemical components, which corroborate the findings of Esmaeel Darezereshki^34^. The DTA shows an endothermic peak at 150 °C with a low weight loss of 0.5 wt% while the second step corresponds to a more significant weight loss of 18 wt% occurring at 300–370 °C, which is due to the combustible organic products present in the prepared sample. The third step revealed another significant weight loss of 5 wt% between the range of 370–440 °C, which is due to the transition phase of synthesized compounds. Finally, DTA shows an endothermic peak at 680 °C, thereafter, the curve becomes parallel to the temperature axis, which emphasizes the high stability of the α-${\text{Fe}}_{2} {\text{O}}_{3}$. It is worthy to note that no associated signal was noticed in the TGA curve when compared with the DTA curve. These confirm the crystallization and phase transition of the α-${\text{Fe}}_{2} {\text{O}}_{3}$ according to Abdelmajid Lassoued et al.^35^. The TG–DTA curves obtained in this study is similar to the pattern shown by Muhammad Waseem et al.^36^.

### Characterizations of the hematite and RBCs

#### FTIR

**Figure 5:**
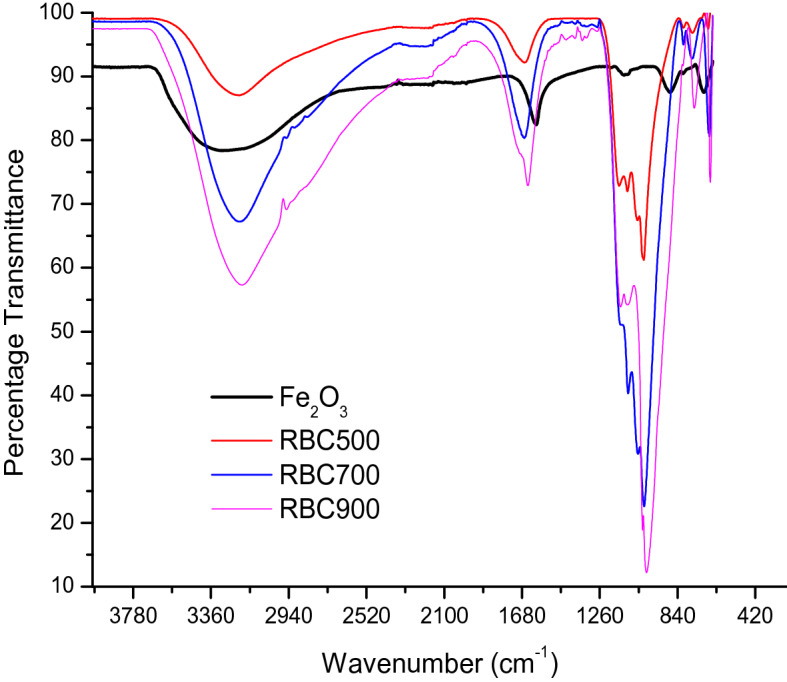
FTIR spectra of the hematite, RBC500, RBC700 and RBC900.

Figure 5 shows the FTIR spectra of the α-${\text{Fe}}_{2} {\text{O}}_{3}$ and RBCs within the range of 4,000–400 cm^−1^. The presence of water (OH) stretch was observed at 3,780–2,910 cm^−1^ region in all the samples. Also, the presence of a peak at 2,950 cm^−1^ can be attributed to C–H stretch. The peak at range 1,200–1,020 cm^−1^ characterized the feature of α-${\text{Fe}}_{2} {\text{O}}_{3}$ which corresponds to the vibration of crystalline Fe–O mode^37^. The S=O symmetric and asymmetric vibration were observed at a wavenumber of 1,100–1,000 cm^−1^ for calcined-sulfonated samples (RBC500, RBC700, and RBC900), which could be as a result of the sulfur anion (${\text{SO}}_{4}^{2 - }$) chelating with iron cation $\left( {{\text{Fe}}^{3 + } } \right)$ on the catalysts^6^. In contrast, α-${\text{Fe}}_{2} {\text{O}}_{3}$ which is un-sulfonated, did not show any band in that regions, indicating the absence of sulfate group. The appearance of two prominent spectra of the four samples from the region of 840–420 cm^−1^ can be attributed to the Fe–O vibration in the rhombohedral lattice of hematite^35^ and the characteristic of the crystalline α-${\text{Fe}}_{2} {\text{O}}_{3}$ compound^38^.

#### XRD

**Figure 6:**
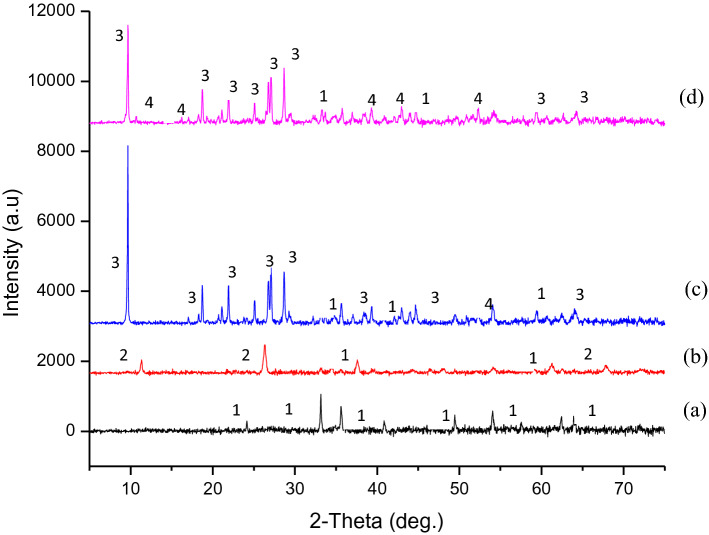
XRD patterns of (**a**) α-${\text{Fe}}_{2} {\text{O}}_{3}$, (**b**) RBC500, (**c**) RBC700, and (**d**) RBC900. Chemical compounds in the products: (1) Hematite—α-${\text{Fe}}_{2} {\text{O}}_{3}$. (2) Aluminum hydrogen sulfate hydrate—${\text{AlH}}\left( {{\text{SO}}_{4} } \right)_{2} \cdot {\text{H}}_{2} {\text{O}}$. (3) Rhomboclase—$\left( {{\text{H}}_{5} {\text{O}}_{2} } \right){\text{Fe}}\left( {{\text{SO}}_{4} } \right)_{2} \left( {{\text{H}}_{2} {\text{O}}} \right)_{2}$. (4) Coquimbite—${\text{Fe}}_{1.68} {\text{Al}}_{.32} \left( {{\text{SO}}_{4} } \right)_{3} \left( {{\text{H}}_{2} {\text{O}}} \right)_{9}$.

The XRD spectra of the α-${\text{Fe}}_{2} {\text{O}}_{3}$ and RBCs are depicted in Fig. 6, in which they all exhibited rhombohedral structure similar to Sivakumar et al.^39^ spectra. The XRD patterns show the crystallinity nature of the compounds present in the samples with definite Bragg’s peak at specific 2θ angles. The peaks appearing at 2θ range of 24.15°, 33.17°, 35.70°, 40.83°, 49.45°, 54.06°, 57.55°, 62.48°, and 63.90° are indexed to hkl (0 1 2), (1 0 4), (1 1 0), (1 1 3), (0 2 4), (1 1 6), (0 1 8), (2 1 4), (3 0 0) respectively, on crystallographic plane of α-${\text{Fe}}_{2} {\text{O}}_{3}$. This is comparable with the findings of Abdelmajid Lassoued et al.^35^. These can be attributed to the crystalline structures corresponding to pure α-${\text{Fe}}_{2} {\text{O}}_{3}$ nanoparticles. The appearance of the highest peak at 33.17° indicates the presence of α-${\text{Fe}}_{2} {\text{O}}_{3}$ according to Muhammad Waseem et al.^36^. While the narrowness of the sharp peaks indicate high crystallinity and high purity of the hematite according to Abdulmajid Lassoued et al.^35^. The hematite obtained in this study, therefore, can be of quality to replace off-the-shelf hematite for applications, such as sensors, catalysts, data storage materials, fine ceramics, pigments, and photo-electrochemical cells^14^. Similar intense peaks were observed for the RBCs, which also justify the high crystallinity of the catalysts. The spectra show majorly, the presence of (1) Hematite—α-${\text{Fe}}_{2} {\text{O}}_{3}$ (JCPDS: 033-0664), (2) Aluminium hydrogen sulfate hydrate—${\text{AlH}}\left( {{\text{SO}}_{4} } \right)_{2} \cdot {\text{H}}_{2} {\text{O}}$ (JCPDS: 027-1006), (3) Rhomboclase—$\left( {{\text{H}}_{5} {\text{O}}_{2} } \right){\text{Fe}}\left( {{\text{SO}}_{4} } \right)_{2} \left( {{\text{H}}_{2} {\text{O}}} \right)_{2}$ (JCPDS: 070-1820), and (4) Coquimbite—${\text{Fe}}_{1.68} {\text{Al}}_{.32} \left( {{\text{SO}}_{4} } \right)_{3} \left( {{\text{H}}_{2} {\text{O}}} \right)_{9}$ (JCPDS: 074-2406). These show that the product at each phase consists of pure compounds. Further identification of compounds represented by the peaks was done using the JCPDS file number as indicated in the bracket. While the mean crystalline size of each of the products was calculated using the Debye–Scherrer’s equation. The results of the crystalline size as shown in Table 2 for α-${\text{Fe}}_{2} {\text{O}}_{3}$, RBC500, RBC700 and RBC900 are 16.43, 9.77, 10.25 and 14.13 nm respectively. Although, the previous report gave an average particle size of α-Fe_2_O_3_ in the range of 30–70 nm^39^. However, in this study, the average particle size of α-${\text{Fe}}_{2} {\text{O}}_{3}$ has been calculated as 16.43 nm. The difference between these values might be due to the difference in the process conditions such as agitation speed and calcination temperature. For the RBCs, it can be observed that the mean crystallite size of RBC500 nanoparticles is the minimum size while that of RBC900 is the maximum size. This signifies that the crystalline size increases with increasing calcination temperature which is the same pattern reported by Gaber et al.^40^.

**Table 2:** Crystalline size, average particle size, surface area, pore-volume and pore diameter of the products.

Sample	Crystallite size (XRD) (nm)	Average particle size (nm)	Surface area (BET) (m^2^/g)	Pore volume (DFT) (cc/g)	Pore diameter (DR) (nm)
α-${\text{Fe}}_{2} {\text{O}}_{3}$	16.43	304.4	107.1	0.541	9.236
RBC500	9.77	259.6	434.9	0.144	5.740
RBC700	10.25	169.5	457.5	0.153	5.766
RBC900	14.13	95.62	471.3	0.160	5.842

### SEM/EDS and PSD analyses

**Figure 7:**
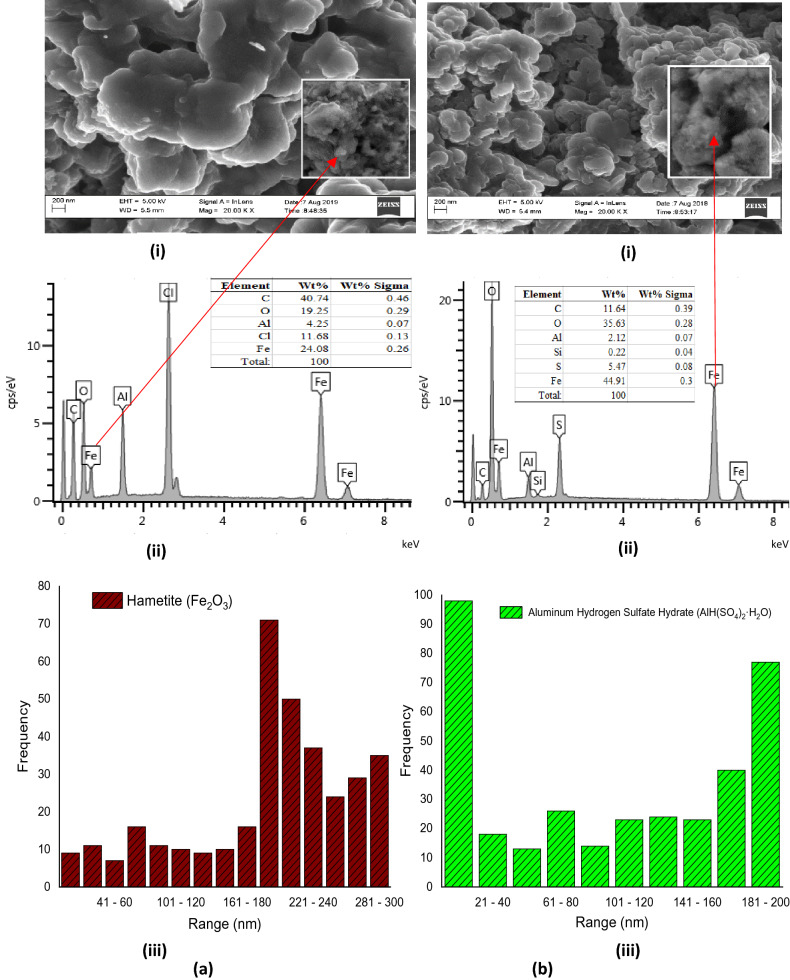

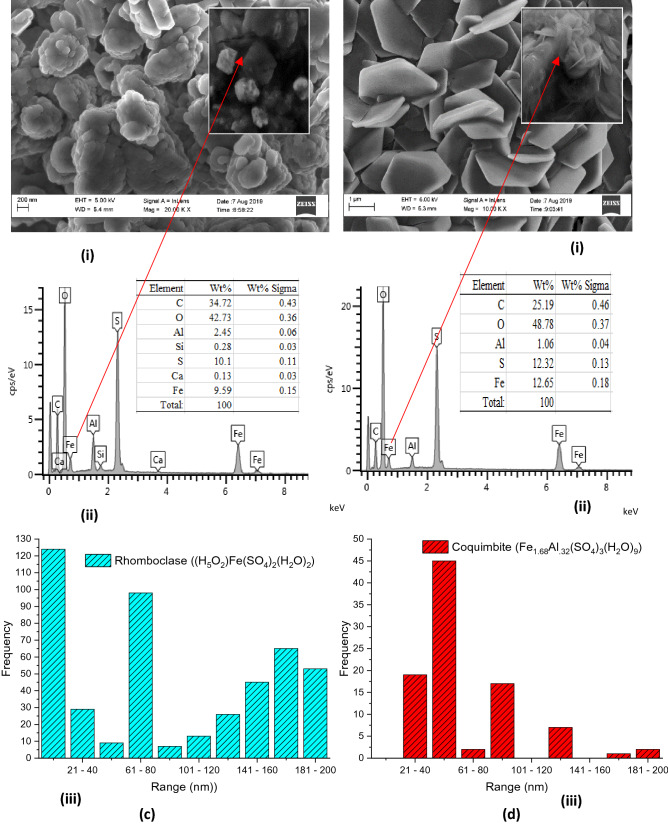
(**a**,**b**) (i) SEM, (ii) EDX and (iii) PSD of WIF to Synthesize (**a**) Fe_2_O_3_ (300 nm PS), and (**b**) RBC500 (200 nm PS). Figure 7 (**c,d**) (i) SEM, (ii) EDX and (iii) PSD of WIF to Synthesize (**c**) RBC700 (200 nm PS), and (**d**) RBC900 (200 nm PS).

The SEM/EDS analyses studied include morphology, elemental composition, and particle size distributions (PSD) as shown in Fig. 7a(i–iii)–d(i–iii). Figure 7a(i) revealed the results of the hematite $\left( {\alpha {\text{-} \text{ Fe}}_{2} {\text{O}}_{3} } \right)$ with the morphology showing clusters of rough surfaces laced with pores and irregular shapes. Figure 7a(ii) is the EDS of the hematite spectrum which shows that the particle consists of ${\text{Fe}}/{\text{Cl}}/{\text{Al}}/{\text{O}}$ with evaluated molar ratio 3/1/0.5/2. The EDS elemental composition confirmed the formation of α-${\text{Fe}}_{2} {\text{O}}_{3}$, especially considering the presence of ${\text{Fe}}^{3 + }$ around the line energy of oxygen (0–2 keV). This is similar to the findings of Nagaraj Basavegowda et al.^14^^,^ where hematite can be closely packed with oxygen lattice. The appearance of sharp peaks between the line energy of 6 and 8 keV in the EDS spectra confirmed the presence of elemental iron^37^. The traces of chlorine were present and may have been introduced possibly during the treatment with ${\text{HCl}}$ while the Al and C originated from the WIF as impurities. Figure 7a(iii) shows the particle size distribution (PSD) of the hematite. The PSD indicates the dominance of particle size range of 181–300 nm, which is in agreement with the SEM morphology as shown in Fig. 7a(i). This finding suggests that the α-Fe_2_O_3_ is a nanoparticle of sub-300 nm.

After calcination at 500 °C and sulphonation by ${\text{H}}_{2} {\text{SO}}_{4}$ of the initial α-${\text{Fe}}_{2} {\text{O}}_{3}$ to obtain RBC500, the morphology reveals less agglomeration with improved smooth surface and pores as shown in Fig. 7b(i). The EDS result in Fig. 7b(ii) shows Fe/S/Al/O with molar ratio 7/1/0.3/5 showing α-${\text{Fe}}_{2} {\text{O}}_{3}$ as a dominant compound with the presence of sulfur. This result revealed that the calcination process removed Cl and that the sulphonation process introduced sulfur compound into the new compound (RBC500). It could also be observed that the PSD was altered possibly due to calcination, and the RBC500 formed exhibited particle size ranges that are rather bi-modal as shown in Fig. 7b(iii). The PSD, however, is evenly distributed in the sub-200 nm which is lower than that of α-${\text{Fe}}_{2} {\text{O}}_{3}$ (sub-300 nm). In addition, the SEM morphology of RBC500 (Fig. 7b(i)) is an indication that the new particle sizes were formed after calcination and sulphonation.

Figure 7c(i–iii) is the morphology, EDS and PSD of the RBC700 respectively. Figure 7c(i) shows that the particles are clustered together and have an ovoid shape with a crystalline surface of the porous structure. The EDS of RBC700 as shown in Fig. 7c(ii) reveals that the Fe/S/Al/O has a molar ratio of 1/1/0.28/4 which indicates that ${\text{Fe}}^{3 + }$ and ${\text{SO}}_{4}^{2 - }$ dominate the sample with traces of Al. The PSD also exhibited a bi-modal structure as shown in Fig. 7c(iii). The figure reveals that the particle sizes are in the sub-200 nm range and are evenly distributed to sub-20 nm in the ranges of 20–200 nm. Among the ranges, sub-20 nm has the highest particles of 125 and next is 61–80 nm with particle numbers of 98. This shows that most of the particles in the RBC700 are sub-100 nm, indicating a nano-catalyst.

Figure 7d(i) shows the morphology of the RBC900, which reveals a well-arranged, smooth, clean and clear crystalline particles. The particles are intercalated and well-aligned rhombohedral centred hexagonal-shaped nano-plates which is one of the characteristics of the hematite compound as reported by Basavegowda et al.^14^. The width of the plates is in microns and is sub-200 nm thick with a well-arranged regular porous structure. The particles are visible and seen as hexagonal crystals with the size varying from 500 nm to 1,000 nm. Figure 7d(ii) shows EDS of the RBC900 as Fe/S/Al/O with molar ratio 1/1/0.08/4 which indicates Al–${\text{Fe}}_{2} {\text{O}}_{3} /{\text{SO}}_{4}$ as the dominant compounds. Figure 7d(iii) reveals the PSD of RBC900, which shows the dominance of a particle size range of 41–60 nm. This result is in agreement with the SEM morphology as shown in Fig. 7d(i) and it suggests that the RBC900 is a nanoparticle of sub-100 nm. Generally, the particle size decreases as the calcination temperature increases. This is similar to the findings of Salam Al-jaberi et al.^6^ which confirms the significant effects of calcination temperature on the nano-synthesis of the catalysts.

### DLS

**Figure 8:**
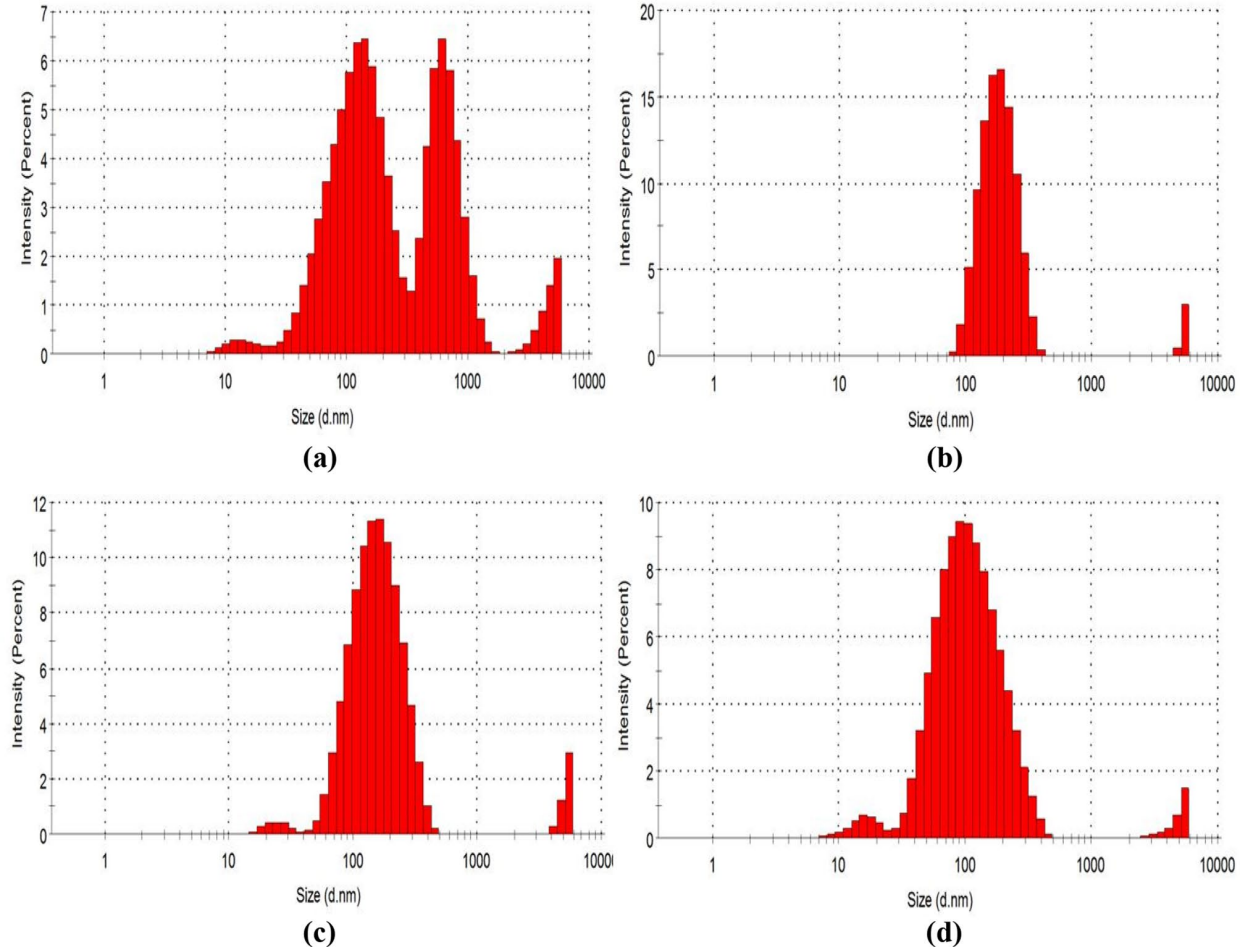
DLS of (**a**) α-${\text{Fe}}_{2} {\text{O}}_{3}$ synthesized from WIF (**b**) RBC500 (**c**) RBC700 and (**d**) RBC900.

The particle size distribution and average particle size of the α-${\text{Fe}}_{2} {\text{O}}_{3}$ and RBCs were analyzed by the DLS and the results obtained are shown in Fig. 8 and Table 2. Figure 8a–d show the particle size distribution of the α-${\text{Fe}}_{2} {\text{O}}_{3}$, RBC500, RBC700 and RBC900 respectively, which follow similar patterns as the PSD. The average particle size of each of the samples as shown in the table are 304.4, 259.6, 169.5 and 95.62 nm for α-${\text{Fe}}_{2} {\text{O}}_{3}$, RBC500, RBC700 and RBC900 respectively. It can be seen from Fig. 8a that the particle size distribution for the α-${\text{Fe}}_{2} {\text{O}}_{3}$ lies between 10 and 1,500 nm. Whereas, Fig. 8b–d shows that the particle size distribution for the RBCs lies between 10 and 400 nm. This suggests that the thermal treatment of the α-${\text{Fe}}_{2} {\text{O}}_{3}$ further reduced the particle size distribution of the RBCs. The RBC900 has the smallest average particles size, followed by RBC700 and the highest is RBC500 as shown in the table. Thus, as the calcination temperature increased, the nanoparticle size decreases which confirms the results of the PSD. The results show that all the RBCs have an average particle size less than 300 nm and also reveals that there is a significant improvement to the particle size based on the calcination temperature between 500 and 900 °C. This shows that each of the RBCs has more loading sites for the transesterification process, as the smaller the average particle size of the solid catalyst, the larger the surface area, which makes more loading sites available for catalytic activity. The nanoparticle size is also directly proportional to the magnetophoretic forces (Fmag). Since the average particle size of the RBCs is between 90 and 260 nm, hence, the Fmag is sufficient to overcome both thermal randomization energy and viscous hindrances of the transesterification reaction^41^. The findings from the DLS corroborates the PSD of the SEM which confirms that the solid acid catalysts in this study are nanocatalysts in nature.

### BET

The catalytic activity of any solid catalyst has been reported to depend on its surface area, pore-volume, and pore diameter. Hence, the more the surface area (SA), pore volume (PV), and pore diameter (PD), the more the catalytic activity of the catalyst in the process^42^. In this study, the SA for α-Fe_2_O_3,_ RBC500, RBC700 and RBC900 are 107.1, 434.9, 457.5 and 471.3 m^2^/g respectively, PV; 0.541, 0.144, 0.153 and 0.160 cc/g respectively and PD; 9.236, 5.740, 5.766 and 5.842 nm respectively as shown in Table 2. The parameters obtained for the α-${\text{Fe}}_{2} {\text{O}}_{3}$ are very close to those reported by Muhammad Waseem et al.^36^ and this further confirms that the WIF is suitable to produce pure α-${\text{Fe}}_{2} {\text{O}}_{3}$. Furthermore, the RBCs have a higher SA and lower PV compare to the α-${\text{Fe}}_{2} {\text{O}}_{3}$ with increasing calcination temperature and sulphonation process. This indicates that the RBCs would favor good dispersion of active centers and provide mass-transfer advantages^43^. Mostafa Feyzi et al.^44^ reported that the catalytic activity of the solid catalyst is directly dependent on SA, PV and PD. It was reported that the pore structure is a basic requirement for an ideal solid catalyst for biodiesel production. This is because a typical triglyceride molecule has a PD of approximately 5.8 nm. The larger PD (5.740, 5.766, and 5.842 nm) obtained for the RBCs and larger interconnected pores of the triglyceride molecule (5.8 nm) would minimize diffusion limitations of reactant molecules^44^. However, the PD of the RBCs fall within the range of mesoporous (2–50 nm), an indication of excellent catalytic activity^45^. Meanwhile, the mesoporous catalyst has been reported for biodiesel production^44^. The SA of the catalysts is about 4 times higher than the α-${\text{Fe}}_{2} {\text{O}}_{3}$ (107.1 m^2^/g). This result shows the formation of the highly porous structure of the RBCs that can be attributed to the small crystal size as obtained in the XRD analysis^37^. Hence, the high PV would facilitate the reaction by amplifying the reaction surface^44^. The crystalline size of the samples as shown in Table 2 further confirms the nanoparticle size of the α-${\text{Fe}}_{2} {\text{O}}_{3}$ and RBCs. Therefore, the large surface area, pore-volume, and mesoporous structure of the catalysts would make them excellent in the catalytic property^37^.

### Acidity and basic strength of the α-${\text{Fe}}_{2} {\text{O}}_{3}$ and RBCs

In transesterification reaction, the acidity of solid acid catalyst is a crucial factor, as the acidic sites activate the carbonyl groups of triglycerides to initiate the catalytic process^31^. Also, the alkalinity of the catalyst is another important factor that influences the transesterification activity, as the more the alkalinity of the catalyst, the better the biodiesel yield^43,44^. As such, absolute values for the surface coverages of the acidic and basic sites of the α-${\text{Fe}}_{2} {\text{O}}_{3}$ and RBCs were determined through acid–base titrations and the results are presented in Table 3. From the table, α-${\text{Fe}}_{2} {\text{O}}_{3}$ contains 2.21 ± 0.02 mmol g^−1^ acid value which is very close to 1.89 mmol g^−1^ reported by Wenlei Xie et al.^8^. It should be noted that few acid sites were detected on the α-${\text{Fe}}_{2} {\text{O}}_{3}$. The low acidic site of α-${\text{Fe}}_{2} {\text{O}}_{3}$ may not be sufficient to catalyze the trans-esterification reaction^8^. However, the RBC500, RBC700 and RBC900 contain the highly acidic site of 7.84 ± 0.1, 8.92 ± 0.05 and 10.77 ± 0.1 mmol g^−1^ respectively. These values show that the RBCs possess high acidic site concentrations between 7.8 and 10.7 mmol g^−1^ and compare well with those of the sulfonated solid superacid catalysts reported in the literature^46^. But higher than the value of 1.18 mmol g^−1^ reported by Jabbar Gardy et al.^47^ and 0.32 mmol g^−1^ obtained for Fe^3+^/SO_2_ catalyst^21^. The higher acidic values obtained for the RBCs were due to the addition of sulfate groups in the form of SO_2_ on the catalyst surface^19^. The acidity of the RBCs confirmed that the sulfonic acid groups covalently attached onto α-${\text{Fe}}_{2} {\text{O}}_{3}$ to produce stable solid superacid catalysts. Therefore, the acidic properties of the RBCs can significantly improve the catalytic activity of the solid acid catalysts toward the trans-esterification reaction of WCO to biodiesel^8^. In addition, the α-${\text{Fe}}_{2} {\text{O}}_{3}$, RBC500, RB700 and RBC900 also possess basic site of 8.14 ± 0.6, 6.36 ± 0.2, 5.89 ± 0.4 and 4.90 ± 0.2 mmol g^−1^ respectively. All the samples exhibited high basic strength with the α-${\text{Fe}}_{2} {\text{O}}_{3}$ having the highest basic sites. The basic strength of the RBCs show a decreasing trend with respect to the addition of ${\text{SO}}_{4}^{2 - }$ loading. The acidic centres of ${\text{SO}}_{4}^{2 - }$ submerged in the basic matrix of the α-${\text{Fe}}_{2} {\text{O}}_{3}$ resulting in concomitant acidic–basic centers^43^.

**Table 3:** Acid–base characterization of the α-${\text{Fe}}_{2} {\text{O}}_{3}$ and RBCs.

Sample	Acid sites (As) (mmol g^−1^)	Base sites (Bs) (mmol g^−1^)
α-${\text{Fe}}_{2} {\text{O}}_{3}$	2.21 ± 0.02	8.14 ± 0.6
RBC500	7.84 ± 0.1	6.36 ± 0.2
RBC700	8.92 ± 0.05	5.89 ± 0.4
RBC900	10.77 ± 0.1	4.90 ± 0.2

Therefore, the high acid–basic sites obtained for the RBCs reveal the better catalytic activity of the catalysts. As it would be easy for the triglyceride and methanol to diffuse into the interior of the RBCs and contact with more acid–basic active sites^44^.

### Proposed reaction mechanism of the RBCs for biodiesel production

**Scheme 1:**
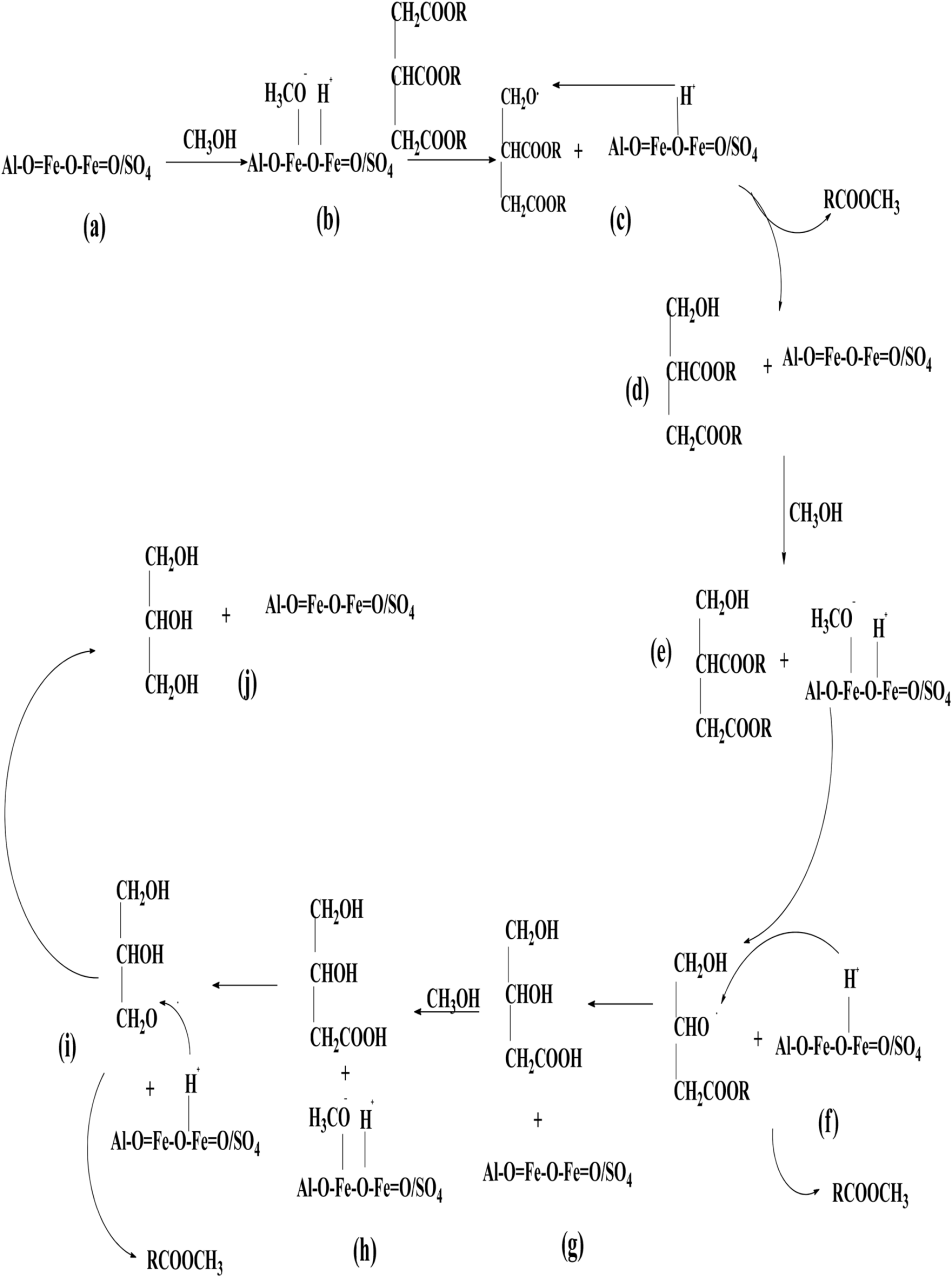
Proposed mechanism for the trans-esterification reactions on *Al* – *Fe*_2_*O*_3_/*SO*_4_. From Scheme 1, item (a) is the RBCs active chemical compound that was developed from calcination and sulphonation of α-${\text{ Fe}}_{2} {\text{O}}_{3}$ synthesized from WIF. Generally, a reaction catalyzed by mineral acid and metal ions such as Al–O=Fe–O–Fe=O/SO_4_ generates hydrogen ion (H^+^) in the initiation step through the protolysis of the ${\text{CH}}_{3} {\text{OH}}$ as shown in item (b). The next stage of the reaction is the protonation of the carbonyl group in the triglyceride by the metal ions in the solid acid catalyst as shown in item (c) to form methyl ester and a diglyceride anion as shown in item (d). This cycle is repeated from item (e) to (f), forming another methyl ester and a monoglyceride anion as shown in item (g). It is repeated the third time as shown in items (h) and (i) to form another methyl ester and glycerol as shown in item (j). From each cycle, Al–O=Fe–O–Fe=O/SO_4_ component of the RBCs is recovered, which is a good characteristic of solid catalysts.

Worthy of note is that the transesterification reaction of triglyceride to produce biodiesel occurs on the surface of the solid catalyst. The RBCs in this study are solid acid catalysts which are composed of Al–O=Fe–O–Fe=O/SO_4_. Meanwhile, Suyin Gan et al.^48^ classified ferric sulfate as a solid acid catalyst that is sparingly soluble in methanol and completely soluble in water. It was also assumed that the reaction of ferric sulphate to produce biodiesel is a pseudo-homogeneous route as shown in Scheme 1. The scheme is the reaction of triglyceride with methanol in the presence of solid acid catalyst developed in this study, where all the chemical compounds involved in the reaction are identified as items (a–j).

### The functionality of the RBCs

**Figure 9:**
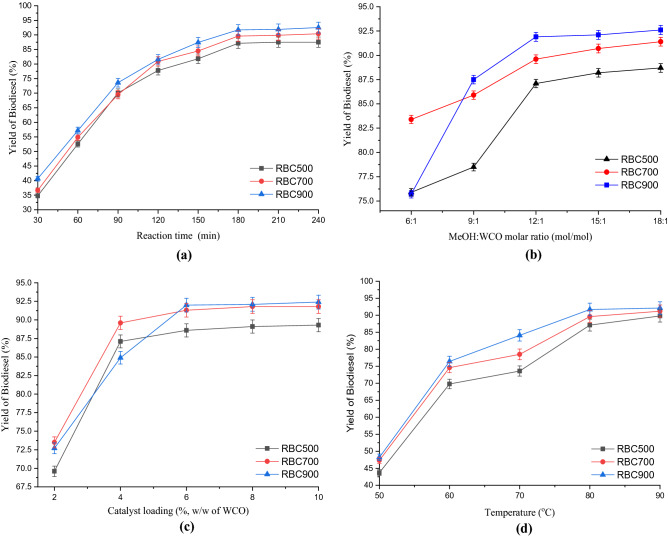
Effects of (**a**) reaction time, (**b**) MeOH: WCO molar ratio, (**c**) catalyst loading and (**d**) reaction temperature on the %yield of biodiesel using synthesized RBC500, RBC700 and RBC900.

The catalyst types of the RBC500, RBC700 and RBC900 were evaluated for their functionality through simultaneous esterification and transesterification of WCO to produce biodiesel. Optimal process conditions were explored by evaluating the effects of reaction time, MeOH: WCO molar ratio, catalyst loading and reaction temperature on biodiesel yield. Figure 9a shows the effect of reaction time between 30 and 240 min at constant MeOH: WCO molar ratio of 1:6, catalyst loading of 10 wt%, reaction temperature of 70 °C and agitation speed of 800 rpm. The %yield of biodiesel increased linearly to about 70% with the time, 90 min for the RBCs, before reaching a plateau after 180 min, to 87.1, 89.6 and 91.7% for RBC500, RBC700 and RBC900 respectively. At the end of the 240 min reaction time, maximum %yield of biodiesel attained was 87.5, 90.4 and 92.5% for RBC500, RBC700 and RBC900 respectively. The MeOH: WCO molar ratio is an important process condition for the transesterification and this is due to the reversibility nature of the reaction. Therefore the %yield of biodiesel can be increased by using excess methanol to favor the forward reaction^8^. The effect of MeOH: WCO molar ratio on the catalytic performance of the RBCs were studied at the range of 6:1 and 18:1 mol/mol under process conditions of reaction time = 3 h, catalyst loading = 10 wt%, reaction temperature = 70 °C and agitation speed = 800 rpm. As seen in Fig. 9b, the biodiesel yield increased significantly as the MeOH: WCO molar ratio increases from 6:1 to 12:1 which is because higher methanol concentrations promote oil solubility and hence %yield of biodiesel^47^. Further increase in the MeOH: WCO molar ratio from 12:1 to 15:1 and 18:1 yielded a marginal increase in the biodiesel yield. Therefore, the appropriate MeOH: WCO molar ratio for the transesterification reaction is considered as 12:1. However, the excess methanol is recyclable through a simple distillation method^8^. The effect of catalyst loading between the range of 2 and 10 wt% on the yield of biodiesel was subsequently explored keeping other process conditions constant (MeOH: WCO molar ratio = 12:1, reaction time = 3 h, reaction temperature = 70 °C and agitation speed = 800 rpm). The result obtained is as shown in Fig. 9c^,^ when the catalyst loading increased from 2 to 6 wt%, the biodiesel yield increased steadily to 88.6, 91.3 and 92.0% for RBC500, RBC700 and RBC900 respectively. The direct proportionality of the catalyst to biodiesel yield can be associated with the increase in the number of active sites to ensure attainment of equilibrium within a shorter time^43^. Additional catalyst loadings (8 and 10 wt%) show an insignificant impact on the %yield of biodiesel for all the RBCs. This is an indication that the trans-esterification is reaction-rate limited for catalyst loading of ≤ 6 wt% and that higher catalyst loading is therefore undesirable^47^. The excess catalyst loading may have enhanced the viscosity of the reaction mixture and hindered the effective mass transfer of the solid catalyst and feedstocks, consequently leading to an insignificant impact to increase the biodiesel yield^8^. Figure 9d shows the effect of reaction temperature at the range of 50 and 90 °C on the %yield of biodiesel for the RBCs under constant reaction conditions (MeOH: WCO molar ratio = 12:1, reaction time = 3 h, catalyst loading = 6 wt% and agitation speed = 800 rpm). As can be clearly seen, the %yield increased continuously as the temperature increases from 60 to 80 °C. This, however, corroborates the claim that the higher temperature accelerates the trans-esterification reaction rate due to the shift of reaction equilibrium and as such, increase the reactant activation, higher oil miscibility and lower the viscosity of reactants^8,47^. Further increase in the reaction temperature to 90 °C reveals negligible impact on biodiesel yield which indicates that the reaction had attained equilibrium. Thus, the reaction temperature of 80 °C can be chosen as the suitable temperature for higher biodiesel yield in this study. Generally, prolonged reaction time of 180 min, excess MeOH: WCO molar ratio of 12:1, catalyst loading of 6 wt% and a higher reaction temperature of 80 °C are the process conditions required to achieve a satisfactory biodiesel yield of 87, 90 and 92% for RBC500, RBC700 and RBC900 respectively. This phenomenon shows that RBC900 with higher SA, PV and PD with the smallest average particle size has a better catalytic performance than the other catalysts. Mostafa Feyzi et al.^44^ observed the same trend in their study.

Therefore, the maximum %yield of biodiesel obtained in this study confirms the functionality of the WIF-based solid acid catalysts which is composed of Al–Fe_2_O_3_/SO_4_. The %yield of biodiesel follows the order of acidity and alkalinity for respective catalyst, therefore the surface acidity was responsible for the high catalytic activity^31^. Jabbar Gardy et al.^47^ in their study of SO_2_/Fe–Al–TiO_2_ catalyst developed from ammonium hydroxide (28–30%, NH_4_OH) and ferric chloride hexahydrate (≥ 98%, FeCl_3_·6H_2_O) as precursors, attained 95.6% yield of biodiesel. Mostafa Feyzi et al.^44^ also obtained a 94.8% yield of biodiesel with their developed catalysts of Cs/Al–Fe_3_O_4_ from the pure ferric compound as a precursor. Comparatively, the solid acid catalyst developed from WIF achieved a satisfactory yield of biodiesel, just as catalysts derived from a pure ferric compound did.

### Reusability test of the RBCs

**Figure 10:**
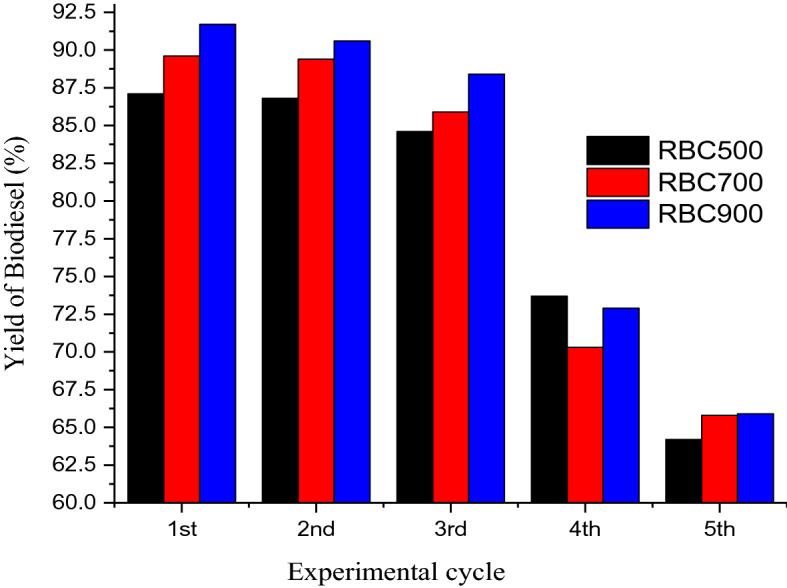
The reusability plot for the RBC500, RBC700 and RBC900.

Figure 10 shows the variation in the %yield of biodiesel with the reuse time for each of the RBCs. As it is observed, no significant loss was noticed in the %yield of biodiesel over the first three consecutive catalytic trans-esterifications, an indication of excellent reusability of the catalysts. However, there was a significant loss in the yield of biodiesel at the fourth cycle to about 72% and the fifth cycle to 65%. The reason for the loss of activity could be associated with the deposition of carbonaceous or organic substrates on the recovered catalyst^31^. Also, ferric sulphate catalyst though insoluble in oil, it is sparingly soluble in methanol and can dissolve in the water formed during esterification^48^. This is a usual phenomenon encounter in the use of heterogeneous catalysts particularly in the polar reaction system that leads to a drastic loss of catalytic activity^31^.

### Characterization of WCO and biodiesel samples produced by the RBCs

#### Physico-chemical properties

**Figure 11:**
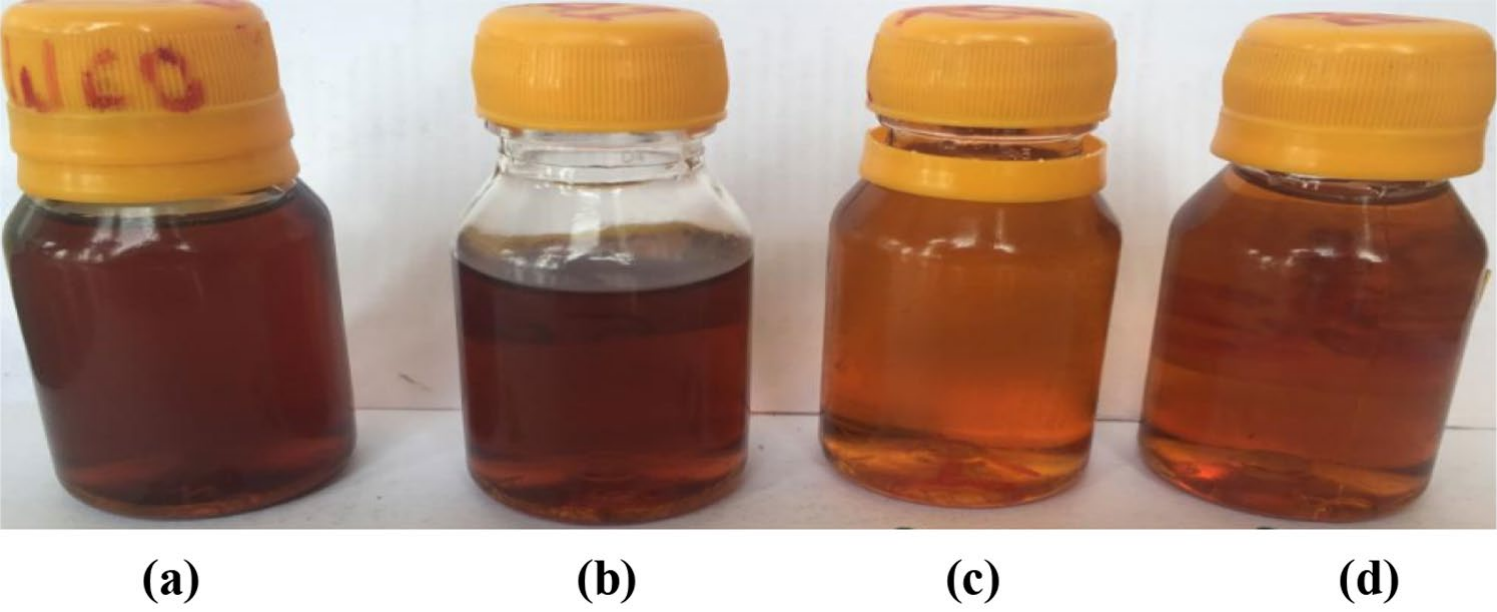
(**a**) WCO, biodiesel produced by (**b**) RBC500, (**c**) RBC700, and (**d**) RBC900 catalysts.

The catalyst type of RBCs was studied for biodiesel production using WCO. The WCO and biodiesel samples obtained from each of the RBCs are shown in Fig. 11. The figure show a color deviation of the biodiesel samples by each of the RBCs from the color of the WCO. The fuel properties obtained by each of the RBCs and the physicochemical properties of the WCO are shown in Table 4 in comparison with the ASTM standards. The values of density obtained are 0.951, 0.868, 0.887 and 0.891 g/cm^3^ at a temperature of 15 °C for WCO, RBC500, RBC700 and RBC900 respectively. The densities of biodiesel from respective catalysts fall within the standard with a significant deviation from that of the WCO. The results show that the RBCs are suitable to produce appropriate biodiesel for use in compression ignition engines (CIE) since the density is an important parameter that determines the energy content of biodiesel.

**Table 4:** Fuel properties of WCO, and biodiesel from WCO by RBC500, RBC700 and RBC900.

Properties (units)	WCO	RBC500	RBC700	RBC900	ASTM limits and testing procedure
		Biodiesel from WCO			
Density (g/cm^3^, 15 °C)	0.951	0.868	0.887	0.891	0.82–0.90 D 4,052
Kv (mm^2^/s, 40 °C)	25.6	4.1	3.3	3.9	1.9–6.0 D 445
Flash point (°C)	159	137	132	130	≥ 130 D 93
Cloud point (°C)	+ 23	+ 7	+ 5	+ 5	− 3 to 12 D 2,500
Pour point (°C)	+ 15	+ 3	− 1	− 1	− 15 to 10 D 97
Cetane number	30.51	56.49	58.74	64.34	> 47 D613

Kinematic viscosity (Kv) evaluates the degree of atomization of the biodiesel in the combustion chamber of CIE. The Kv obtained as shown in Table 4 are 25.6, 4.1, 3.3 and 3.9 mm^2^/s at a temperature of 40 °C for WCO, RBC500, RBC700 and RBC900 respectively. The Kv of the biodiesel samples fall within the range of the standard and this gives an assurance of quality biodiesel that can combust completely in a CIE without leaving residual residues that can cause damage in the engine^49^. Whereas, Kv of the WCO is about one-sixth far higher than that of the biodiesel samples, an indication of the effect of the trans-esterification process.

Flash point (FP) is an important property that has a direct positive relationship with fluid’s viscosity and it is the tendency to form a flammable mixture in the air^50^. The FP is the minimum temperature when there is enough concentration of evaporated fuel in the air for the flame to propagate after ignition has been initiated^51^. As shown in Table 4, the biodiesel samples fall within the standard flash point of ≥ 130 ^o^C. According to Adewale Folayan et al.^50^, FP determines the transportation and storage requirement of biofuel and should be higher than the standard to ensure safe operation and reduced vaporization within the maximum operating temperature. The findings here show that the biodiesel samples are safe for transportation, storage, and handling in safe operation.

Cloud Point (CP) is the temperature at which the cloud of wax crystals become visible in liquid fuel. This is observed when the fuel is cooled under a controlled environment using the ASTM procedure (ASTM D 2500)^49^. The CP values of the biodiesel samples obtained are shown in Table 4 as + 7, + 5 and + 5 for RBC500, RBC700 and RBC900 respectively. These values are relatively low compared to the WCO with the CP of + 23 (Table 4). The CP values of biodiesel are of quality, indicative of great performance and good characteristics of fuel in low-temperature climate conditions. This shows that the biodiesel would have a good fuel flow with great fuel pump, filter and injector performances^50^. On the other hand, pour point (PP) is the lowest temperature when the liquid fuel ceases to flow or be pumped having solidified to resist flow. It is also the dynamic behavior of fuel under varying temperature conditions^51^. For this work, the PP values of biodiesel obtained are shown in Table 4 as + 3, − 1 and − 1 for RBC500, RBC700 and RBC900 respectively. The values are very low compared to the PP of WCO that is + 15, which is an indication of improved quality due to the catalytic reaction of WCO to biodiesel. The values of PP for the different biodiesel suggest that the fuel types do not have inferior cold flow property as the high value of PP can cause gum formation, crystallization of fuel particles and negatively affect fuel flow and ultimately destroy pump and injector^50^.

Cetane number (CN) is the characteristics that reveal the ignition quality of a diesel engine fuel as the higher the cetane number, the shorter the delay time (ID), and the better the ignition quality^51^. Meanwhile, the minimum value of CN recommended by the ASTM standard is 47 (Table 4). The values of the CN obtained for the biodiesel types are 56.49, 58.74, and 64.34 as shown in Table 4, for the RBC500, RBC700 and RBC900, respectively. These values are greater than the minimum standard as well as the CN of WCO (30.51). The high CN in this study may be due to an increasing chain length, increasing branching, and increasing saturation in the fatty acid chain of the biodiesel samples as a result of the trans-esterification by the RBCs^52^. Based on the standard, the biodiesel samples in this work have good ignition quality^53^.

### FAME analysis

**Figure 12:**
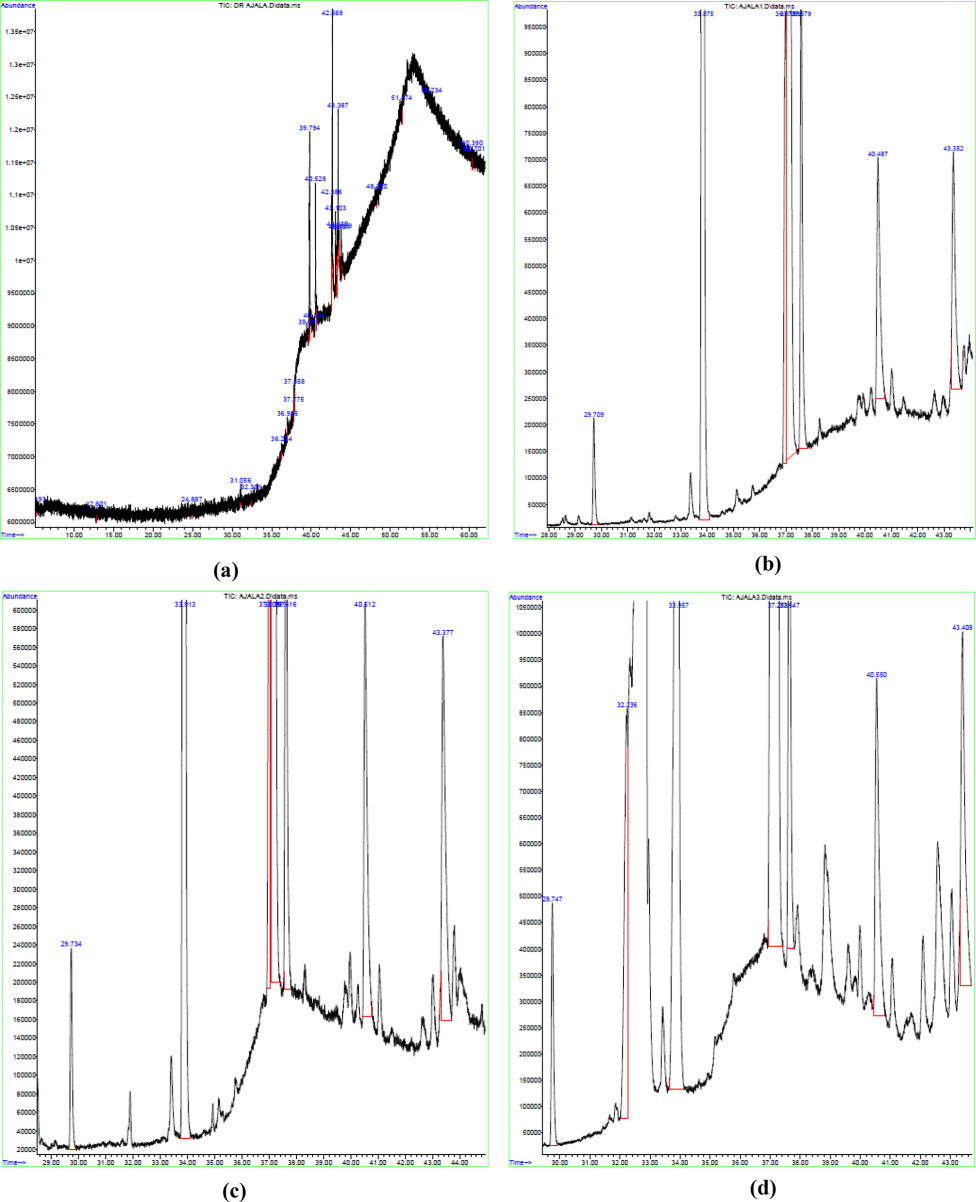
GC–MS spectra of the (**a**) WCO, biodiesel samples by (**b**) RBC500, (**c**) RBC700 and (**d**) RBC900.

The FAME profile and percentage composition obtained from the GC–MS for WCO and biodiesel samples synthesized by the RBCs are shown in Table 5. The spectra for each of the samples were identified with the NIST MS database as shown in Fig. 12a–d for the WCO and biodiesel samples by the RBC500, RBC700 and RBC900 respectively. From the results obtained, the WCO consists of two FAMEs with their respective percentage composition which includes ${\text{C}}_{17} {\text{H}}_{34} {\text{O}}_{2}$ (16.70%) and ${\text{C}}_{19} {\text{H}}_{36} {\text{O}}_{2}$ (20.78%) as shown in the table. However, after trans-esterification of WCO with the RBCs, 4 components were identified with their respective percentage compositions, which include ${\text{C}}_{7} {\text{H}}_{12} {\text{O}}_{4}$, ${\text{C}}_{17} {\text{H}}_{34} {\text{O}}_{2}$, ${\text{C}}_{19} {\text{H}}_{34} {\text{O}}_{2}$ and ${\text{C}}_{19} {\text{H}}_{36} {\text{O}}_{2}$ as shown in the table. The biodiesel sample produced by the RBC500 possesses higher concentrations of ${\text{C}}_{17} {\text{H}}_{34} {\text{O}}_{2}$ (43.20%) and ${\text{C}}_{19} {\text{H}}_{36} {\text{O}}_{2}$ (38.19%), and lower concentrations of ${\text{C}}_{7} {\text{H}}_{12} {\text{O}}_{4}$ (11.38%) and ${\text{C}}_{19} {\text{H}}_{34} {\text{O}}_{2}$ (5.81%)$.$ While the RBC700 and RBC900 biodiesel samples present a similar pattern of percentage compositions of the FAME. Based on the FAME analysis, saturated and unsaturated chains present in the biodiesel samples are 43.20 and 55.38% (RBC500), 43.97 and 54.65% (RBC700) as well as 43.51 and 56.48% (RBC900) respectively. This revealed that the biodiesel samples consist of a higher percentage of unsaturated than saturated fatty acids. The major source of saturated FAME in the samples is methyl palmitate while that of the unsaturated FAME is methyl octadecenoate. The high concentration of unsaturated methyl esters (> 54%) in all the biodiesel samples could be responsible for low values of flash point and cloud point obtained in this work^49^. Furthermore, the high presence of saturated chains (> 43) in the biodiesel samples is responsible for the high cetane number and increased kinematic viscosity recorded in this work^54^. The presence of > 54% unsaturated methyl esters in all the biodiesel samples produced show a high oxidation and thermal stability since the rate of oxidation is on the high side with the increase in the unsaturated fatty acid chains^55^. This however, reveals that the biodiesel samples would have a much slower deterioration rate in high-temperature environments and provides long-term storage duration^56^.

**Table 5:** FAME profile and biodiesel yield of WCO by RBC500, RBC700, and RBC900.

Peak no.	FAME profile	Molecular formula	WCO	RBC500	RBC700	RBC900
				Biodiesel from WCO		
1	Methyl hemiadipate	${\text{C}}_{7} {\text{H}}_{12} {\text{O}}_{4}$	–	11.38	11.21	10.39
2	Methyl palmitate*	${\text{C}}_{17} {\text{H}}_{34} {\text{O}}_{2}$	16.70	43.20	43.97	43.51
3	Methyl linoleate	${\text{C}}_{19} {\text{H}}_{34} {\text{O}}_{2}$	–	5.81	6.18	5.34
4	Methyl octadecenoate	${\text{C}}_{19} {\text{H}}_{36} {\text{O}}_{2}$	20.78	38.19	37.26	40.75
5	None FAME	–	81.23	1.43	1.64	–
	% Saturated FAME		16.70	43.20	43.97	43.51
	% Unsaturated FAME		20.78	55.38	54.65	56.48
	%FAME content^a^		37.47	98.58	98.62	99.99

*Saturated FAME, ^a^Yield (FAME content): >96.5% for ASTM D6751 Standard

The density and kinematic viscosity of the biodiesel samples also depends on the higher unsaturated chain. It is worthy to note that the RBC500, RBC700 and RBC900 produced high FAME contents of 98.22, 98.57 and 99.99% respectively from the WCO as shown in Table 5. The biodiesel yields meet the standard quality of biodiesel as expressed by the ASTM D6751 ( > 96.5%)^32^. Therefore, the biodiesel samples produced by the RBC500, RBC700 and RBC900 are of high quality as they satisfy the ASTM standard.

## Conclusion

This study shows that the WIF is suitable to produce high purity α-Fe_2_O_3_ that can successfully replace off-the-shelf hematite for any application including catalyst development. The solid acid catalysts developed showed great quality suitable for biodiesel production. Their chemical compositions revealed the presence of hematite (α-Fe_2_O_3_) and aluminium hydrogen sulfate hydrate (AlH(SO_4_)_2_·H_2_O) in the RBC500, scanty hematite (α-Fe_2_O_3_), dominant rhomboclase (H_5_O_2_)Fe(SO_4_)_2_(H_2_O)_2_ and scanty coquimbite (Fe_1.68_Al_.32_(SO_4_)_3_(H2O)_9_) in the RBC700. Whilst the RBC900 is composed of scanty hematite (α-Fe_2_O_3_), dominants rhomboclase (H_5_O_2_)Fe(SO_4_)_2_(H_2_O)_2_ and coquimbite (Fe_1.68_Al_.32_(SO_4_)_3_(H2O)_9_). The study concludes that the WIF can produce quality hematite suitable to synthesize the kinds of solid acid catalysts in the presence of sulphuric acid for biodiesel production. While further characterization confirmed the nano-particle nature of the catalysts that results in high efficiency of biodiesel production.

The functionality of the catalysts shows high performance with > 90% yield of biodiesel. The physico-chemical characterization of the biodiesel samples gave a good account that meets the ASTM standard for biodiesel. The FAME profile in the biodiesel samples confirmed that the RBC500, RBC700 and RBC900 are suitable to synthesize reliable and viable alternative fuel for compression ignition engines.
